# Iowa’s Action

**Published:** 1903-04

**Authors:** 


### IOWA'S ACTION.

THE action of the Iowa State Veterinary Medical Association in summarily expelling one of their members who was found guilty of conducting a so-styled "Veterinary Correspondence School," should be a wholesome lesson to those who, under the guise of educating men, make fools of such who are inveigled by their glittering advertisements; ruin others by making quacks and impostors of them, and lead them from quiet, wholesome, pastoral lives to the seductive field of the knave and fraud, and ultimately leave them stranded upon the shores of poverty and disgrace. While all this is going on the measure of loss and injury to an ignorant but confiding public is incalculable, and the distress and suffering that follow in their train from ignorance and deceit, in accepting responsibilities they are unable to discharge, can only be feebly conjectured. Now that Iowa has dealt a stinging blow to this fake institution, may we not ask our Canadian brethren, "What are you going to do with your school of like character?"

---

WE hope to be able in the May number of the JOURNAL to review at some length the legislation that has been enacted in many of the States during the recent legislative sessions. Much progress along wholesome lines has been accomplished, and we are sure that our readers are all interested in the good work.

---

MONTANA has created by legislative enactment the positions of Chief and Assistant State Meat and Milk Inspectors at salaries respectively of $2000 and $1500 annually, and fixed by the same act the requirements of those eligible to these positions. Happily for the good of this legislation, those filling these positions must be qualified veterinarians.

